# Fever of Unknown Origin Attributable to Haematocolpos Infected with *Salmonella enterica* Serotype Typhi Resistant to Nalidixic Acid: A Case Report

**DOI:** 10.3329/jhpn.v31i3.16833

**Published:** 2013-09

**Authors:** Sonal Saxena, Mayank Dwivedi, Priyam Batra, Renu Dutta

**Affiliations:** Department of Microbiology, Lady Hardinge Medical College, New Delhi 110 001, India

**Keywords:** Fever of unknown origin, Haematocolpos, NARST, India

## Abstract

The prevalence of nalidixic acid-resistant *Salmonella* Typhi (NARST) infection is increasing worldwide. We are reporting an unusual case of infected haematocolpos presenting as urinary obstruction in a patient with fever of unknown origin (FUO). This case report highlights the importance of quinolone-resistant typhoid fever in the differential diagnosis of any acute febrile illness in countries, like India, where *Salmonella* infection is endemic.

## CASE REPORT

A 12-year old girl with a 10-hour history of dull lower abdominal pain and inability to void was admitted at the emergency of SSK Hospital attached to Lady Hardinge Medical College, New Delhi. She also gave a history of burning micturition for the last two days and of fever for one month. The fever was low-grade, intermittent, arose at any time of the day without chills or notable sweats, was relieved by medication but recurred. The patient had not attained menarche and was not taking any medications other than acetaminophen.

Her total leukocyte count was 1.7×10^9^/l (neutrophils 51%, lymphocytes 43%, and monocytes 5%). The patient had sterile reports of blood and urine cultures performed within 5 days of the start of fever. On admission, urine and blood culture**s** were repeated but were found sterile. Thin and thick film examinations of peripheral blood were also negative for malaria. Widal test was performed which showed a titre of 80 against ‘O’ (somatic) antigen and 160 against ‘H’ (flagella) antigen of *Salmonella* Typhi (recommended cutoff titres in our hospital is ≥1:80 and ≥1:160 for the ‘O’ and ‘H’ antigens respectively).

On examination, she was in pain, apyrexic with normal pulse rate and blood pressure. On abdominal examination, tenderness was elicited in the midline of the abdomen between the symphysis pubis and two inches below the umbilicus. A distinct suprapubic mass was felt in this location through the abdominal wall, which was symmetrical, tender, and dull to percussion. Rectal examination showed an excessively tender, large, soft swelling bulging into the anterior rectal wall. Vulval examination revealed a tense bulging membrane of bluish discolouration, and haematocolpos was diagnosed.

The patient was catheterized and approximately 500 mL of urine was drained. Hymenectomy with haematocolpos drainage was planned for the patient, and she was admitted for the same.

The patient experienced multiple spikes of fever during the hospital stay and was investigated for the same. She was put on empiric treatment with ciprofloxacin 500 mg intravenously given 12-hourly and injection metronidazole 400 mg given 8-hourly. This was initiated to provide coverage for anaerobic bacteria and facultative anaerobic Gram-negative bacteria, which are common pathogens in such closed-cavity infections, particularly of the genitourinary system.

There was no remission in the patient's temperature, and ceftriaxone was started parenterally on Day 4 of admission. The patient's condition improved, and she had surgery within 24 hours of the initiation of therapy with ceftriaxone. The haematocolpos was drained, and the blood sample was sent for culture. *Salmonella enterica* serotype Typhi was isolated in pure culture from the drained haematocolpos. The isolate was presumptively identified by colony morphology, Gram reaction, motility, and biochemical characteristics as per standard guidelines (1). The isolate was confirmed by slide agglutination with *Salmonella* antisera (Remel Europe Ltd) in accordance with the Kauffman White scheme. By using Vi phage typing according to the International Federation for Enteric Phage Typing, the strain was classified as *Salmonella enterica* serotype Typhi Vi deranged phage type. Antimicrobial susceptibility testing was performed by the disc diffusion test for ciprofloxacin (5 µg), ampicillin (10 µg), cotrimoxazole (1.25/23.75 µg), chloramphenicol (30 µg), nalidixic acid (30 µg), and ceftriaxone (30 µg) as per Clinical Laboratory and Standard Institute guidelines of 2011 (2). The isolate was susceptible to all antibiotics, except nalidixic acid. The antimicrobial susceptibility was also performed using VITEK 2^(R)^ AST card. The organism was reported to be sensitive to amoxycillin-clavulanic acid, ticarcillin, piperacillin, cefpodoxime, cefotaxime, ceftizoxime, aztreonam, meropenem, tetracycline, and tigecycline and resistant to nalidixic caid, norfloxacin, and moxifloxacin. The minimum inhibitory concentration (MIC) of nalidixic acid was ≥32 μg/mL; MIC of moxifloxacin was ≥8 μg/mL, and that of norfloxacin was ≥16 μg/mL.

Enteric fever, an invasive infection consisting of bacteraemia with minimal gastrointestinal infection, is caused by *Salmonella enterica* serotype Typhi and Paratyphi A. Serotype Typhi-related bacteraemia is commonly associated with extra intestinal disease involving liver, spleen, lymph nodes, skin, bones, joints, endocardium, or central nervous system. A review of literature shows that *Salmonella enterica* serotype Typhi is capable of abscess formation in the spleen, liver, and brain. However, patients with these abscesses had associated bacteraemia. Penetration of the extra-intestinal tissues in isolation is rare (3).

Haematocolpos itself is a very uncommon condition and is usually a consequence of cryptomenorrhoea, in which the formation of menstrual discharge goes on without normal appearance of menstruation (4). The isolation of *Salmonella enterica* serotype Typhi from haematocolpos blood without systemic bacteraemia is an extremely rare finding. Transient bacteraemia associated with inadvertent self-administration of antimicrobial agents for fever prior to admission could be a possible explanation for the incongruous observation. This is quite possible in the Indian setting where over-the-counter purchase of antibiotics is a common practice.

The isolate in this study was sensitive to most of the antibiotics, except quinolones. This is unusual but can be explained by the fact that resistance to other antibiotics is plasmid-mediated and is independent of the resistance to fluoroquinolones, which is chromosomally mediated (5). The therapeutic failure by empirical quinolone therapy, as observed in the case, highlights the emerging problem of antimicrobial resistance in *Salmonella enterica* serovar Typhi and the therapeutic dilemma faced by the clinicians regarding the empirical use of fluoroquinolones for the treatment of acute febrile illnesses.

### Conclusions

This case report documents the isolation of *Salmonella* Typhi from haematocolpos blood without systemic bacteraemia. Also, the susceptibility pattern of the isolated organism highlights the emerging problem of antimicrobial resistance in serovar Typhi and the therapeutic dilemma faced by the clinicians regarding the empirical use of fluoroquinolones.
